# Supplementary material for: Is retroflexion a stable cue for distributional learning for speech sounds across languages? Learning for some bilingual adults, but not generalisable to a wider population in a well powered pre-registered study
Source: PeerJ. 2023 Jul 10;11:e15467. doi: 10.7717/peerj.15467 (PMC10340096; doi:10.7717/peerj.15467)
Supplement: Supplemental Information 1 [file peerj-11-15467-s001.pdf]

Q490 This information is allows us to better understand your answers.

We will not collect identifying or sensitive information and your answers will be stored securely.

Q1347 What is your nationality?

____

Q1348 What country did you mostly grow up in, during your pre-school (0-5 years old)?

____

Q1349 What country did you mostly go to school in, for your junior years (6-12 years old)?

____

Q1350 What country did you mostly go to school in for your high-school years (13-18 years old)?

____

Q442 What country have you mostly lived in for the past 3 years

____

Q1351 If you have lived in other countries for one year or more, please list them below:

____

____

____

____

____

Q1336 For each of your languages (including dialects), tell us how good you are at... *Understanding when people speak*
7 = native-level, 1= a few words, 0=no knowledge

	(1)	(2)	(3)	(4)	(5)	(6)	(7)	I don't understand this language (0)
English (1)	○	○	○	○	○	○	○	○
Mandarin Chinese (2)	○	○	○	○	○	○	○	○
Tamil (3)	○	○	○	○	○	○	○	○
Malay (4)	○	○	○	○	○	○	○	○

Q1337 Are there other languages/dialects that you understand when people speak?

○ Yes (1)

○ No (2)

Display This Question:

If Q1337 = Yes

Q1338

	(1)	(2)	(3)	(4)	(5)	(6)	(7)	Don't Understand (0)
1. (please name) (1)	○	○	○	○	○	○	○	○
2. (please name) (2)	○	○	○	○	○	○	○	○
3. (please name) (3)	○	○	○	○	○	○	○	○
4. (please name) (4)	○	○	○	○	○	○	○	○

Display This Question:

If If <o:p></o:p> 1. (please name) Is Displayed

Q1339 Add more?

○ Yes (1)

○ No (2)

Display This Question:

If Q1339 = Yes

Q1340

	(1)	(2)	(3)	(4)	(5)	(6)	(7)	Don't Understand (0)
5. (2)	○	○	○	○	○	○	○	○
6. (3)	○	○	○	○	○	○	○	○
7. (4)	○	○	○	○	○	○	○	○
8. (5)	○	○	○	○	○	○	○	○

Q1341 For each of your languages (including dialects), tell us how good you are at... *Speaking to others*

7 = native-level, 1= a few words, 0=no knowledge

	(1)	(2)	(3)	(4)	(5)	(6)	(7)	I don't speak this language (0)
English (1)	○	○	○	○	○	○	○	○
Mandarin Chinese (2)	○	○	○	○	○	○	○	○
Tamil (3)	○	○	○	○	○	○	○	○
Malay (4)	○	○	○	○	○	○	○	○

Q1342 Are there other languages/dialects you can speak?

- Yes (1)
- No (2)

Display This Question:

If Q1342 = Yes

Q1343

	(1)	(2)	(3)	(4)	(5)	(6)	(7)	Don't speak (0)
1. (1)	○	○	○	○	○	○	○	○
2. (2)	○	○	○	○	○	○	○	○
3. (3)	○	○	○	○	○	○	○	○
4. (4)	○	○	○	○	○	○	○	○

Display This Question:

If If 1. Is Displayed

Q1344 Add more?

- Yes (1)
- No (2)

Display This Question:

If Q1345 = Yes

Q1345

	(1)	(2)	(3)	(4)	(5)	(6)	(7)	Don't understand (0)
5. (2)	○	○	○	○	○	○	○	○
6. (3)	○	○	○	○	○	○	○	○
7. (4)	○	○	○	○	○	○	○	○
8. (8)	○	○	○	○	○	○	○	○
