# Supplementary figures and images for: Is retroflexion a stable cue for distributional learning for speech sounds across languages? Learning for some bilingual adults, but not generalisable to a wider population in a well powered pre-registered study
Source: PeerJ. 2023 Jul 10;11:e15467. doi: 10.7717/peerj.15467 (PMC10340096; doi:10.7717/peerj.15467)

**PCA\_screepplot**

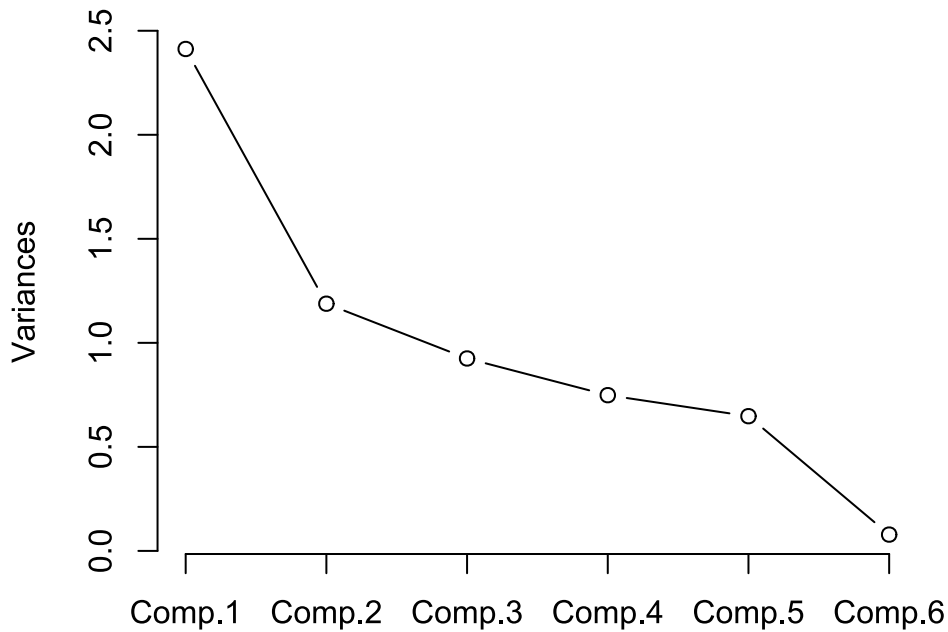

Supplement: Supplemental Information 2 [file peerj-11-15467-s002.pdf]
